# Comparative Evaluation of Apparent Diffusion Coefficient Values of White Matter Surrounding the Heterotopia in Children With Unilateral Subependymal Heterotopia

**DOI:** 10.7759/cureus.21458

**Published:** 2022-01-20

**Authors:** Yesim Eroglu, Kevser Tuncer Kara

**Affiliations:** 1 Department of Radiology, Firat University School of Medicine, Elazig, TUR; 2 Department of Public Health, Firat University School of Medicine, Elazig, TUR

**Keywords:** children, white matter, apparent diffusion coefficient (adc), diffusion-weighted imaging, subependymal heterotopia

## Abstract

Introduction: To compare the apparent diffusion coefficient (ADC) values of the white matter around heterotopia in children with unilateral subependymal heterotopia with those of the symmetrical normal cerebral hemisphere and control group.

Methods: Between January 2011 and September 2021, 15 pediatric patients with unilateral focal subependymal heterotopia among 47 patients with heterotopia detected in brain magnetic resonance imaging (MRI) in our hospital were included in the study. The control group consisted of 15 age- and sex-matched children with normal neurological examination and normal brain MRI. In brain MRIs, ADC value was measured from the white matter around the heterotopia area and from the opposite cerebral hemisphere matched to the location, and from the bilateral location-matched white matter of the control group. The area of heterotopia was measured on axial T1-weighted MRI. The data were evaluated statistically.

Results: There were eight girls and seven boys in the heterotopia group. The median age was 5.00 (min: 3, max: 14). There was no statistically significant difference between the ADC values of the heterotopia side and contralateral white matter of the heterotopia group. In addition, no statistically significant difference was found between the heterotopia side and opposite sides of the heterotopia and control groups ADC values.

Conclusion: According to the findings of this study, no difference was found in the ADC values of the white matter around the lesion in children with subependymal heterotopia compared to the opposite cerebral hemisphere and control groups.

## Introduction

Subependymal heterotopia (SEH) is the most common subtype of gray matter heterotopia. It consists of gray matter clusters located under the ependyma of the lateral ventricles [1]. SEH can be unilateral focal, bilateral focal, and bilateral diffuse. Gray matter heterotopia is a cortical malformation that develops as a result of the interruption of the migration of neurons in the brain to the cortex during the fetal period. It consists of normal neuron clusters located in abnormal localization in the brain as a result of a disorder in the migration of neurons [2,3].

Magnetic resonance imaging (MRI) is the main imaging method used in the diagnosis of heterotopia foci with high soft-tissue resolution. Heterotopia is located in abnormal localizations on brain MRI and is seen as isointense foci with the gray matter on all sequences. Gray matter heterotopia is divided into three groups as subependymal, subcortical, and band heterotopia according to the location and shape of heterotopic neurons in MRI [4,5]. There are different opinions that these neurons, which are abnormally localized in patients with heterotopia, lose their direction during migration and go in the wrong direction or migrate too far to the subpial area. In addition, there are different opinions about whether heterotopia clusters form abnormal interconnections in the brain and whether they cause abnormal trophic effects on the surrounding tissue with the normal appearance or limited connections with other areas of the brain. In the literature, there are many different opinions about the anomalies that can be seen in the normal-appearing white matter, deep gray matter, and the interconnections between them in patients with gray matter heterotopia [6].

Diffusion-weighted imaging (DWI) quantitatively measures the microscopic diffusion of water molecules in biological tissues, giving an idea of the functional data of the tissue [6-8]. In the literature, it has been reported that DWI has important contributions to conventional MRI by showing the abnormality in the white matter that appears normal [9,10]. The aim of this study was to compare the apparent diffusion coefficient (ADC) values of white matter around heterotopia in children with unilateral focal SEH with those of symmetrical normal cerebral hemisphere and control group.

## Materials and methods

Formation of patient and control group

The study was approved by the non-interventional research ethics committee of our university (2021/10-32/23.09.2021). Images of patients whose brain MRIs were reported as heterotopia in our hospital between January 2011 and September 2021 were analyzed. A total of 47 pediatric patients whose brain MRI was reported as heterotopia was found in the archives of our hospital. By examining the images of these patients, patients with subcortical and band heterotopia, patients with unilateral or bilateral diffuse and bilateral focal SEH, and patients with accompanying cerebral anomalies were excluded from the study. In addition, in order for the measurements to be homogeneous, two patients with unilateral focal SEH only on the right side of the brain were excluded from the study, and 15 pediatric patients with unilateral focal SEH only on the left side were included in the study. The control group consisted of 15 age- and sex-matched children with normal neurological examination and normal brain MRI.

Image acquisition and analysis

**Figure 1:**
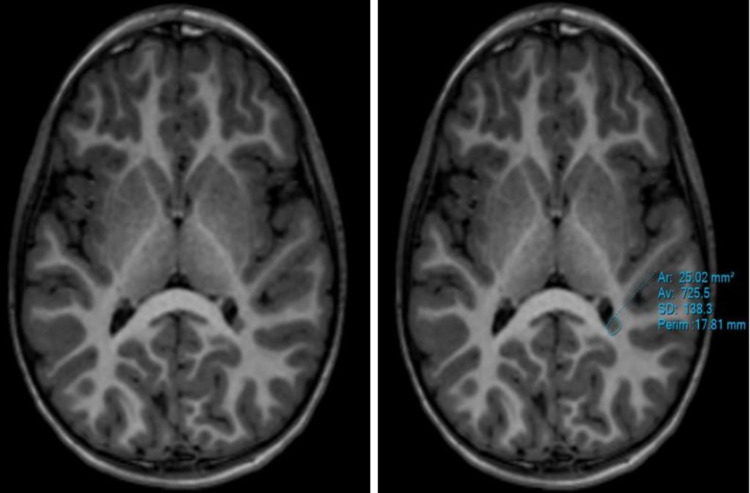
Axial T1-weighted MRI Measurement of the area of the subependymal heterotopia located at the level of the posterior horn of the left lateral ventricle was done.

**Figure 2:**
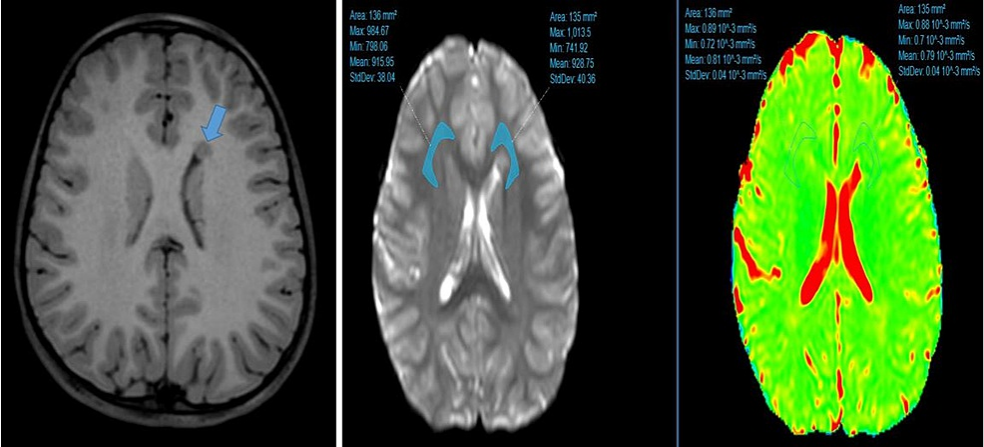
Axial T1-weighted MRI showed subependymal heterotopia in the left frontal DWI showed ADC measurement from the white matter around the heterotopia focus and from the opposite cerebral hemisphere. DWI: diffusion-weighted imaging; ADC: apparent diffusion coefficient

Brain MRI of the patients was performed on 1.5 and 3 Tesla MR devices in our hospital (Philips Healthcare, Ingenia, Netherlands). Time echo = 80 ms, time repetition = 3200 ms, field of view (FOV) = 230 mm, matrix = 256 × 256 mm, section thickness = 5 mm, b value = 0-1000 mm^2^/s parameters were used to obtain the DWI sequence. The heterotopia area was measured in the axial 3D T1-weighted Turbo field echo image (Figure 1). The ADC value was measured by placing a single region of interest (ROI) in the white matter adjacent to the heterotopia focus in such a way that it surrounds the heterotopia focus. The mean ROI size was 150.20 ± 28.39 mm^2^. Care was taken to place the ROI only in the white matter. ROI was localized to exclude the heterotopia focus and cerebral cortex (Figure 2). The ADC value was also measured from the white matter of the symmetrical normal cerebral hemisphere by replicating the same ROI at the workstation. Measurements were made from the same localizations in the control group.

Statistical analysis

SPSS 22.0 package program (IBM SPSS Statistics for Windows, Version 22.0. Armonk, New York) was used in the statistical analysis of the study. Non-parametric tests were used because the number of cases was 15. Percentage and median (min-max) values were given in the descriptive findings according to the characteristics of the variables. The Wilcoxon test was used to compare the ADC values, and the Spearman correlation test was used to evaluate the correlation of numerical data. A p-value of <0.05 was considered statistically significant.

## Results

There were eight girls and seven boys in the heterotopia group. The median age was 5.00 (min: 3, max: 14). Seven (46.6%) had epilepsy, four (26.6%) had headache, two (13.4%) had neuromotor developmental delay, one (6.7%) had speech retardation, and one (6.7%) had applied with the complaint of physical growth retardation. The lesion size, ADC values of the heterotopia group, and ADC values of the control group are given in Table 1.

**Table 1:** Size of heterotopia and ADC values of heterotopia and control groups ADC: apparent diffusion coefficient

		ADC values			
	Heterotopia group			Control group	
	Size of heterotopia (mm^2^)	Contralateral hemisphere (Right)	Heterotopia side (Left)	Right hemisphere	Left hemisphere
Median	34.68	0.80	0.80	0.80	0.80
Minimum	14.36	0.69	0.65	0.72	0.71
Maximum	71.90	0.93	0.92	0.94	0.94

There was no significant difference between the ADC values of the heterotopia side (left) and the opposite side (right) of the heterotopia group (p>0.05). In addition, no significant difference was found between the ADC values of the heterotopia side and the opposite side of the heterotopia and control groups (p>0.05), (Table 2, Wilcoxon test).

**Table 2:** Comparison of ADC values ADC: apparent diffusion coefficient; SEH: subependymal heterotopia

ADC values	p	Z
SEH Left-Right	0.689	-0.400
Control Left-Right	0.740	-0.332
SEH Left-Control Left	0.649	-0.455
SEH Right-Control Right	0.861	-0.175

Considering the correlations between age, heterotopia size, and ADC values; as the age increased, the contralateral ADC values of the heterotopia group and the right and left ADC values of the control group decreased (p=0.41, 0.41, 0.019). In the heterotopia group, the ADC value of the heterotopia side and the ADC value of the contralateral side showed a strong positive correlation (p=0.00, r=0.884). The right and left ADC values of the heterotopia and control groups were also positively correlated (Table 3, Spearman Correlation test).

**Table 3:** Correlation of variables r = correlation coefficient; p = significance value *Correlation is significant at the 0.05 level (2-tailed) **Correlation is significant at the 0.01 level (2-tailed)

		Age	Size	Heterotopia side	Contralateral side with heterotopia patient	Control Right	Control Left
Age	r	1	0.369	-0.352	-0.532*	-0.532*	-0.594*
	p		0.175	0.198	0.041	0.041	0.019
Size	r		1	-0.386	-0.321	0.183	0.225
	p			0.155	0.244	0.544	0.419
Heterotopia side	r			1	0.884**	0.502	0.520*
	p				0.000	0.057	0.047
Contralateral side with heterotopia patient	r				1	0.550*	0.577**
	p					0.034	0.024
Control right	r					1	0.956**
	p						0.000
Control left	r						1
	p						

## Discussion

The aim of this study was to compare the ADC values of the white matter around heterotopia in children with unilateral SEH with those of the symmetrical normal cerebral hemisphere and control group. In this study, there were eight girls and seven boys in the heterotopia group. The median age was 5.00 (min: 3, max: 14). There was no statistically significant difference between the ADC values of the heterotopia side and contralateral white matter of the heterotopia group. In addition, no statistically significant difference was found between the heterotopia side and opposite sides of the heterotopia and control groups ADC values.

The results of this study differ from the literature. In studies in the literature, it has been determined that there is an increase or decrease in some diffusion values of perilesional white matter in children with periventricular nodular heterotopia. In this study, however, no significant difference was found in the ADC values of the white matter around heterotopia in children with SEH.

Gray matter heterotopia is a cortical developmental disorder that results from the failure of neurons to migrate from the periventricular area to the cortex. Gray matter clusters are seen in localizations that should not be in the brain. The incidence of epilepsy is very high in patients with gray matter heterotopia [11-15]. The most common subtype is SEH. The pathogenesis of heterotopia has not been fully explained, and there are different views on the inadequate migration of neurons, as well as the fact that neurons lose their direction during migration and go in the wrong direction, or migrate too far to the subpial area. In addition, there are different opinions as to whether heterotopia clusters cause abnormal interconnections in the brain and whether they cause abnormal trophic effects on surrounding tissue with the normal appearance or insufficient connections with other parts of the brain [3,5,16].

DWI is an MRI technique that measures the random movement of water in biological tissues to gain insight into the microstructure of tissues [17]. It gives functional information about the cell density of tissues [18]. The first use of DWI was the detection of acute ischemia [19]. However, over the years, while it has had an important place in clinical imaging, especially in recent years, it has also become the focus of scientific research. Diffusion is the free movement of water molecules. Since this movement in biological tissues will be limited by the cell membrane and molecules, this diffusion movement in biological tissues is defined as ADC. It has been mentioned in some studies that DWI can show anomalies in white matter that appear normal in other MRI sequences [9,10]. Increased ADC values suggest that at this level of the brain, the extracellular space and water level increase, and the amount of cells decreases. In addition, this may suggest the presence of abnormal myelination [20]. Briganti et al. performed brain diffusion tensor imaging (DTI) of a newborn with bilateral SEH. They found that fractional anisotropy decreased and axial diffusivity and radial diffusivity values increased compared to newborns with moderate perinatal asphyxia and normal MRI findings. They thought that this might be due to the change in the intermediate regions of the brain where neuronal migration is stopped in the newborn with SEH [21]. In another study, it was found that fractional anisotropy, which is one of the DTI values of perilesional white matter, decreased, mean and radial diffusivity increased, there was no difference in axial diffusivity in children with periventricular nodular heterotopia compared to controls, and these changes were thought to be related to microstructural changes in the white matter [22]. In our study, no significant difference was found between the ADC values of the white matter adjacent to the heterotopia and the location-matched white matter in the opposite cerebral hemisphere of children with SEH. There was no significant difference in the ADC values of the white matter of the children with SEH compared to the ADC values of the white matter of the control group. In addition, it was found that ADC values decreased in both SEH and control groups in correlation with age. We think that this is a condition that occurs due to the normal myelination process of the brain [23]. This finding may mean that the white matter myelination of children with SEH is not affected.

DTI is a diffusion imaging method that shows the maximum diffusion direction in the medium by measuring the diffusion motion of water molecules in many directions. It is an advanced MRI technique that includes some parameters such as fractional anisotropy, axial diffusivity, mean diffusivity, and radial diffusivity [19,24].

The limitations of our study are the inadequacy of the patient population and the lack of clinical data. It is a single-center study. Despite being screened the last 10 years, our number of cases is low, since patients mostly come from the east of the country. We also recognize that DTI measurements are valuable. However, since our study is a retrospective study and DTI examination was not performed on the patients, the existing DWI examinations were evaluated.

## Conclusions

As a result, in this study, unlike the literature, no difference was found in the ADC values of the white matter around the lesion in children with SEH compared to the opposite cerebral hemisphere and control group. Further multicenter studies need to support this finding.
